# Case Report: A case of traumatic subgaleal hematoma with delayed massive exophthalmos

**DOI:** 10.3389/fsurg.2025.1594871

**Published:** 2025-09-09

**Authors:** Hui Jae Lee, JaeHwan Jung, Taesung Joo, In-Ki Park, Jun Seok Koh, Min Seok Kang, Jae-Ho Shin

**Affiliations:** ^1^ Seoul Ire Eyeclinic, Kyung Hee University School of Medicine, Seoul, Republic of Korea; ^2^ Department of Ophthalmology, Kyung Hee University Hopsital at Gangdong, Kyung Hee University School of Medicine, Seoul, Republic of Korea; ^3^ Department of Ophthalmology, Kyung Hee University Medical Center, Kyung Hee University School of Medicine, Seoul, Republic of Korea; ^4^ Department of Neurosurgery, Kyung Hee University Hopsital at Gangdong, Kyung Hee University School of Medicine, Seoul, Republic of Korea

**Keywords:** compressive optic neuropathy, exposure keratopathy, orbital subperiosteal hematoma, subgaleal hematoma, surgical drainage

## Abstract

Progressive exophthalmos occurring after minor trauma is very rare, it is important to consider subgaleal hematoma in the differential diagnosis. If diagnosis is delayed, permanent vision loss may occur due to optic nerve damage or corneal damage due to pressure, so performing decompression surgery at an appropriate time is effective in preventing blindness. A 16-year-old male patient with Lennox-Gastaut syndrome and developmental disability was admitted to the hospital 2 weeks after a head injury due to increased swelling and ecchymosis of left eyelid and suspicion of compressive optic neuropathy of the left eye due to massive exophthalmos. Visual acuity measurement was not possible due to the patient's condition., and the intraocular pressure in the left eye was 20 mmHg. The pupil size in both eyes was the same, and there was a pupil reflex in the left eye, and there were no abnormal findings in the blood coagulation test. Computed tomography (CT) showed a subperiosteal hematoma in the left orbit and left eye severe proptosis and deviation. To control intraocular pressure and relieve exposure keratopathy, the orbital hematoma was removed through a sub-brow incision, and a lateral canthotomy was performed, and a drain was installed to drain blood accumulated in the orbit under general anesthesia. Orbital CT taken for follow-up observation showed that the hematoma had decreased compared to the day of visit. Regarding the subgaleal hematoma, hematoma was aspirated three times at the neurosurgery department. After surgery, ointments for exposure keratopathy. During follow-up, corneal transparency was maintained and visual acuity was confirmed to be intact by VEP (Visual Evoked Potential).

## Introduction

Subgaleal hematoma is a hemorrhage between the periosteum and the Gallean aponeurosis that occurs when minor scalp trauma causes radial or tangential force to rupture the emissary vein (1). If the bleeding continues, it can extend below the aponeurosis of the occipitofrontalis muscle and invade the subcutaneous tissue of the neck (2). If the bleeding exceeds the supraorbital ridge, it can cause proptosis, decreased vision, diplopia, elevated intraocular pressure, exposure keratopathy and pain (3, 4). Orbital involvement of subgaleal hematoma is relatively rare and has been reported to occur immediately after trauma up to 11 days later (4). It is known that surgical intervention is needed in cases of orbital invasion accompanied by increased intraocular pressure, ophthalmoplegia, exposure keratopathy and visual impairment (5, 6). We report a case of traumatic subgaleal hematoma with delayed massive proptosis with severe exposure keratopathy in which visual acuity and corneal function were preserved through successful treatment.

## Case presentation

**Figure 1:**
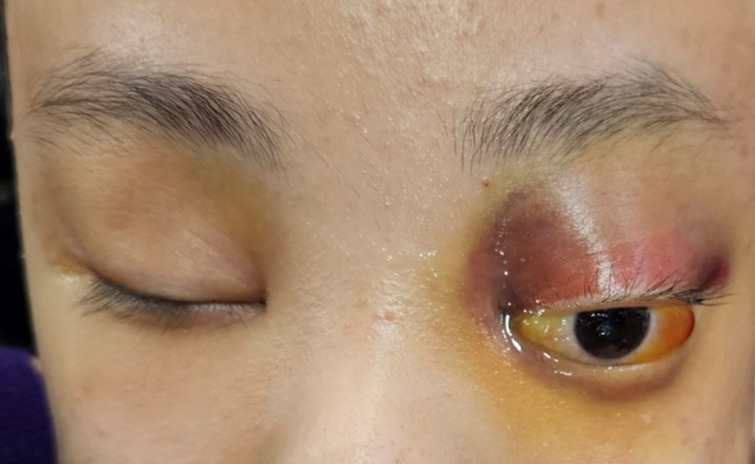
Proptosis, ecchymosis, lagophthalmos and conjunctival chemosis of the left eye.

**Figure 2:**
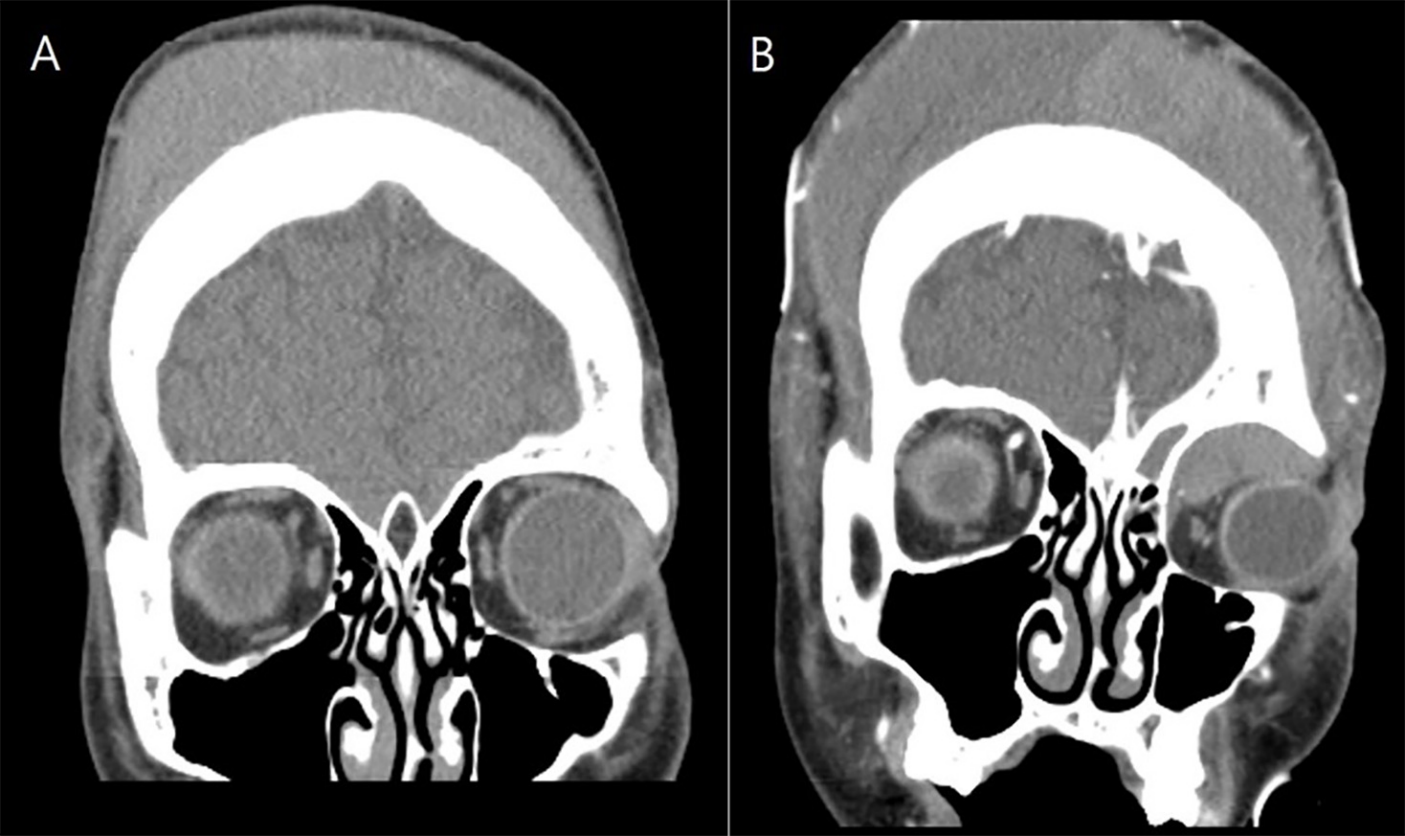
Orbital CT scans. **(A)** CT performed four days before hspitalization. A subgaleal hematoma and swelling near the left orbit were observed, but no intraorbital hematoma was observed. **(B)** CT taken at the time of hospitalization. Subgaleal hematoma was observed in the right frontal, occipital, and left temporal regions. A suspected hematoma was observed in the left intraconal space, and left eye proptosis and deviation became worse compared to 4 days ago.

A 16-year-old male patient with Lennox-Gastaut syndrome and developmental disability was admitted to the hospital two weeks after a head injury due to increased swelling and ecchymosis of left eyelid and suspicion of compressive optic neuropathy of the left eye due to massive exophthalmos (Figure 1). A 16-year-old male patient suffering from Lennox-Gastaut syndrome and developmental disability undergoing follow-up observation at another hospital due to subgaleal hematoma that occurred after an injury to the left head in a bathtub 2 weeks ago. Visual acuity could not be evaluated due to poor cooperation, and intraocular pressure was 16 mmHg in the right eye and 20 mmHg in the left eye. Extraocular muscle movements and eye movement restriction also could not be evaluated and an exophthalmos of 8 mm in the left eye was measured by Hertel exophthalmometry. The left eyelid was tense, erythematous, severe lagophthalmos were observed. In the portable slit lamp examination, severe conjunctival chemosis and congestion were observed in the left eye, especially in the temporal area, and multiple epithelial defects were observed in the exposed cornea due to exposure keratitis. The pupil size in both eyes was the same, and there was a pupil reflex in the left eye, but it was impossible to test for RAPD due to exposure keratopathy and poor cooperation. The left optic disc border was clear and no optic disc pallor was observed. Blood tests were normal and there was no hemorrhagic tendency. CT scans taken at the time of admission showed a suspicious hematoma in the left extraconal space and left eye proptosis and deviation became worse compared to the image taken 4 days ago (Figure 2). Subgaleal hematoma was observed in the right frontal, occipital and left temporal regions. After hospitalization, intraocular pressure was controlled and lubricant ointment and antibiotic ointment were applied to prevent further damage to the cornea due to exposure keratitis. It was decided to perform emergency hematoma aspiration to control intraocular pressure, relieve pressure on the optic nerve, and prevent additional corneal damage due to exposure keratitis. Under general anesthesia, the orbital hematoma was removed through a sub-brow incision, lateral canthotomy was performed, and a drain was installed to drain blood accumulated in the orbit. After surgery, intravenous steroid pulses were administered (500 mg/day, 12 times) and ointment was maintained to prevent corneal opacity.

**Figure 3:**
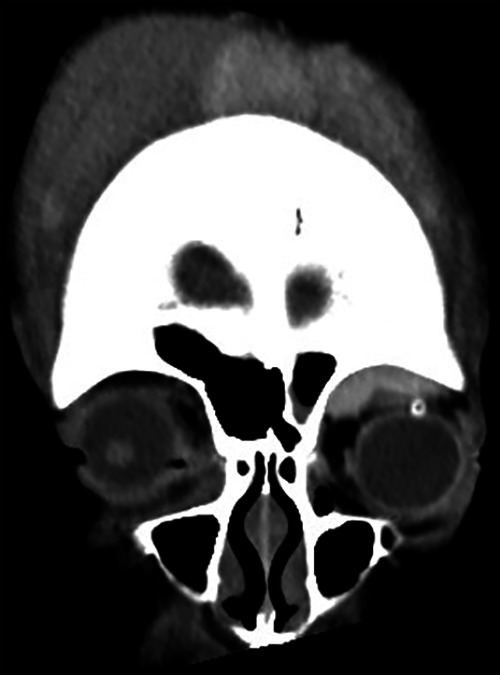
CT performed on the 5th day after surgery showed a large amount of subaponeurotic hemorrhage on the scalp, intraorbital hematoma was significantly reduced compared to the time of admission, and an inserted drain tube was observed.

A follow-up CT performed 5 days after surgery confirmed that the orbital hematoma had decreased (Figure 3). On the 5th, 10th, and 19th days after surgery, subgaleal hematoma aspiration was performed three times in the neurosurgery department, and 300 cc, 200 cc, and 175 cc of blood were drained, respectively, and a compression bandage was maintained on the head. On the 10th day after surgery, the drain was removed because blood was no longer drained, the lubricating ointment (Durateras ointment, Alcon, USA) was maintained, the antibiotic ointment (Ocuflox ointment, Samil, Korea) was changed to a combination ointment (Maxitrol, Alcon, USA) containing steroids, and oral steroids were discontinued. Twenty days after surgery, proptosis and periorbital swelling of left eye had significantly improved, and fix and follow in both eyes were confirmed, the patient was discharged while maintaining ointment. Six months after discharge, the cornea remained transparent and visual function was confirmed by intact flash VEP.

## Discussion

Subgaleal hematoma is caused by relatively minor head trauma and usually improves in most cases with conservative treatment. Possible complications include airway compression, orbital subperiosteal hematoma, infection, and blood transfusion due to hopovolemia (3). Airway compression is caused by excessive hematoma on the face and neck and requires emergency tracheostomy and surgical perfusion to maintain breathing and prevent skin necrosis (2). In the case of orbital subperiosteal hematoma, emergency treatment is necessary because it affects the vision prognosis if orbital compartment syndrome occurs or the cornea is not maintained due to continuous exposure (5, 6).

The exact route by which subgaleal hematoma invades the orbit is not known. Subgaleal hematoma occurs when shear forces destroy blood vessels crossing the loose areolar tissue of the scalp between the Gallia aponeurosis above and the periosteum of the skull below. The frontalis muscle is continuous with facial muscles, including the procerus and orbicularis oculi, without bony attachments. Therefore, blood may drain into the superior orbital ridge and skull base, where the attachment between the Gallia aponeurosis and the periosteum is loose, causing an orbital subperiosteal hematoma. The supraorbital nerve may be another route for orbital hematoma because it exits the orbit through the supraorbital notch or foramen, penetrates the frontal lobe, and remains in the subcostal plane until it enters the frontalis and corrugator muscles (7, 8). According to a summary of 14 cases of orbital involvement reported by Karcioglu ZA et al. (4), the average age was 9.7 years (3.5–18 years), 8 cases were bilateral, and the average time from head trauma to orbital presentation was 5 days (1–11 days). Of these, 11 cases underwent ophthalmological examination, 8 of them had poor visual acuity, 4 cases had severe proptosis and oculomotor paralysis, and all 11 cases received surgical treatment.

Clinical presentation of orbital subperiosteal hematoma include proptosis, decreased vision, diplopia, elevated intraocular pressure, exposure keratopathy and pain (3, 4). Permanent visual loss is caused by uncontrolled intraocular pressure increase due to orbital compartment syndrome or corneal opacity that occurs after exposure keratopathy. Therefore, in the case of orbital subperiosteal hematoma without visual symptoms, conservative treatment is indicated with close observation and steroid therapy, but in cases with visual symptoms, severe exophthalmos, or exposure keratitis, immediate intervention, intraocular pressure control, and aggressive treatment for exposure keratitis are recommended (9).

In addition to aggressive treatment in delayed massive subgaleal hematoma, what is important is evaluation of the presence of accompanying bleeding disorder. Common bleeding disorders include von Willebrand disease, hemophillia A, and hemophillia B, and other reported diseases include sickle cell disease, vitamin C deficiency, hypervascularization, and thrombocytophenia following congenital heart disease. Hematological evaluation to detect these includes complete blood count, prothrombin time, and partial thromboplastin time and in our case, all results were within the normal range (4, 10).

In this case, in which a delayed massive hematoma that occurred 11 days after trauma invaded the orbit, immediate and accurate evaluation of visual function was difficult due to the underlying disease, but aggressive surgical treatment was performed to prevent permanent corneal damage due to exposure keratopathy. After emergent orbital surgical drainage, the patient recovered without any complications by using lubricating ointment, intraocular pressure control, and multiple drainage in neurosurgery. In cases of unilateral exophthalmos in young patients with limited intellectual capacity, it is important to keep in mind the various potential causes and a thorough review is required to ensure an accurate etiological evaluation.

## Data Availability

The original contributions presented in the study are included in the article/Supplementary Material, further inquiries can be directed to the corresponding author.

## References

[1] Kuban K Winston K Bresnan M. Childhood subgaleal hematoma following minor head trauma. Am J Dis Child. (1983) 137(7):637–40. 10.1001/archpedi.1983.02140330021006 6858975

[2] Pagkou D Papavramidis T Mavropoulou X Moysidis M Patsalas I. Massive subgaleal hematoma in a 62-year-old man treated with apixaban as a consequence of mild head trauma. Folia Med. (2021) 63(4):613–7. 10.3897/folmed.63.e58872 35851164

[3] Aubert B Cadoux M Sahyoun C. Traumatic subgaleal hematoma draiage in an adolescent: a case report and review of the literature. Front Pediatr. (2023) 11:1182899. 10.3389/fped.2023.1182899 37325363 PMC10266201

[4] Karcioglu ZA Hoehn ME Lin YP Walsh J. Ocular involvement after subgaleal hematoma. J AAPOS. (2008) 12(5):521–3. 10.1016/j.jaapos.2008.03.004 18620880

[5] Pope-pegram LD Hamill MB. Post-traumatic subgaleal hematoma with subperiosteal orbital extension. Surv Ophthalmol. (1986) 30(4):258–62. 10.1016/0039-6257(86)90122-0 3952648

[6] Pomeranz AJ Ruttum MS Harris GJ. Subgaleal hematoma with delayed proptosis and corneal ulceration. Ann Emerg Med. (1995) 26(6):752–4. 10.1016/s0196-0644(95)70051-x 7492049

[7] Antón J Pineda V Martin C Artigas J Rivera J. Posttraumatic subgaleal hematoma: a case report and review of the literature. Pediatr Emerg Care. (1999) 15(5):347–9. 10.1016/j.inat.2017.04.006 10532668

[8] Kim SY Cha HG Jang SY Hwang SC. Delayed massive expansion of subgaleal hematoma complicated with proptosis in hemophilia B. Korean J Neurotrauma. (2021) 17(2):149–55. 10.13004/kjnt.2021.17.e14 34760826 PMC8558023

[9] Adeleye AO. Subgaleal haematoma extending into orbit following blunt head trauma as a cause of permanent blindness: a case illustrated review. Niger J Ophthalmol. (2017) 25(1):1–5. 10.4103/0189-9171.207372

[10] Zimmerman B Valentino LA. Hemophilia: in review. Pediatr Rev. (2013) 34(7):289–94. 10.1542/pir.34-7-289 23818083

